# Post-traumatic endophthalmitis prophylaxis: a systematic review and meta-analysis

**DOI:** 10.1186/s12348-022-00317-y

**Published:** 2022-11-18

**Authors:** Joshua M. Van Swol, Walter K. Myers, Jonathan A. Beall, Miriam M. Atteya, Jeffrey P. Blice

**Affiliations:** 1 College of Medicine, Medical University of South Carolina, Charleston, SC USA; 2 Department of Public Health Sciences, Medical University of South Carolina, Charleston, SC USA; 3 Department of Ophthalmology-Vitreoretinal Surgery, Medical University of South Carolina, Charleston, SC USA

**Keywords:** Open globe, Post-traumatic, Endophthalmitis, Meta-analysis, Systematic review, Prophylaxis

## Abstract

**Purpose:**

The goal of this study is to determine if certain aspects of endophthalmitis prophylaxis strategies are superior to others.

**Design:**

This investigation is a systematic review and meta-analysis.

**Methods:**

All studies specifying a type of prophylaxis strategy and resulting rates of endophthalmitis were included. Time course, method of administration, and antibiotic regimen, and confounding factors were collected and included for meta-regression.

**Results:**

Time courses greater than 24 h did not significantly improve outcomes. Likewise, intraocular and/or intravenous antibiotic administration methods did not significantly outperform oral administration. No antibiotic regimens performed differently from vancomycin/ ≥ 3^rd^ generation cephalosporin except for ciprofloxacin monotherapy which yielded significantly worse outcomes.

**Conclusions:**

Future antibiotic strategies should strongly consider the risks of antibiotic treatment > 24 h and administration methods other than the oral antibiotic forms. In addition, providers should be wary of using ciprofloxacin monotherapy for endophthalmitis prophylaxis when treating open globe injuries.

**Supplementary Information:**

The online version contains supplementary material available at 10.1186/s12348-022-00317-y.

## Introduction

While treatment strategies have improved, endophthalmitis remains a serious ophthalmologic disease. Endophthalmitis, a purulent inflammatory condition of the eye, can result in catastrophic complications such as reduced visual acuity, blindness, and can even necessitate an enucleation in refractory cases [1]. For this reason, it is considered one of the leading causes of monocular vision loss [2]. Endophthalmitis can occur due to a systemic infection reaching the eyes, known as endogenous endophthalmitis, but most commonly occurs through trauma, surgery, or corneal infections, otherwise called exogenous endophthalmitis [3]. Traumatic endophthalmitis risk factors include intraocular foreign bodies, rural trauma, wound size, lens rupture, and delayed repair [2]. However, a consensus on the most favorable prophylaxis management remains unreached [4]. Finding an optimum prophylaxis regimen could reduce the ocular morbidity related to endophthalmitis. The aim of this study is to evaluate different prophylactic approaches in the literature too discover factors correlated with a lower incidence of endophthalmitis. In this investigation, a comprehensive systematic review was performed to search for studies using standard endophthalmitis prophylaxis protocols after open globe injuries (OGIs) and a meta-regression was performed to evaluate different treatment factors.

## Materials and methods

### Search criteria

This study was done according to the Preferred Reporting Items for Systematic Reviews and Meta-Analyses (PRISMA) guidelines [5]. Detailed search strategies were developed for the following three databases: PubMed (U.S. National Library of Medicine, National Institutes of Health), Cochrane Library (Wiley), and CINAHL (EBSCO). Databases were searched from date of inception through May 18, 2022. The search strategy included the keywords: “endophthalmitis,” “traumatic,” and “open globe”. The PubMed search strategy was reformatted to be used in the two other databases. Additional file 1: Appendix A details the search strategy and results for each database. References of relevant articles were searched to verify the search strategy as well as to include any articles not captured by the search. The review management software Covidence (Veritas Health Innovation Ltd, Melbourne, Australia) was used for study selection.

### Selection criteria

**Fig. 1:**
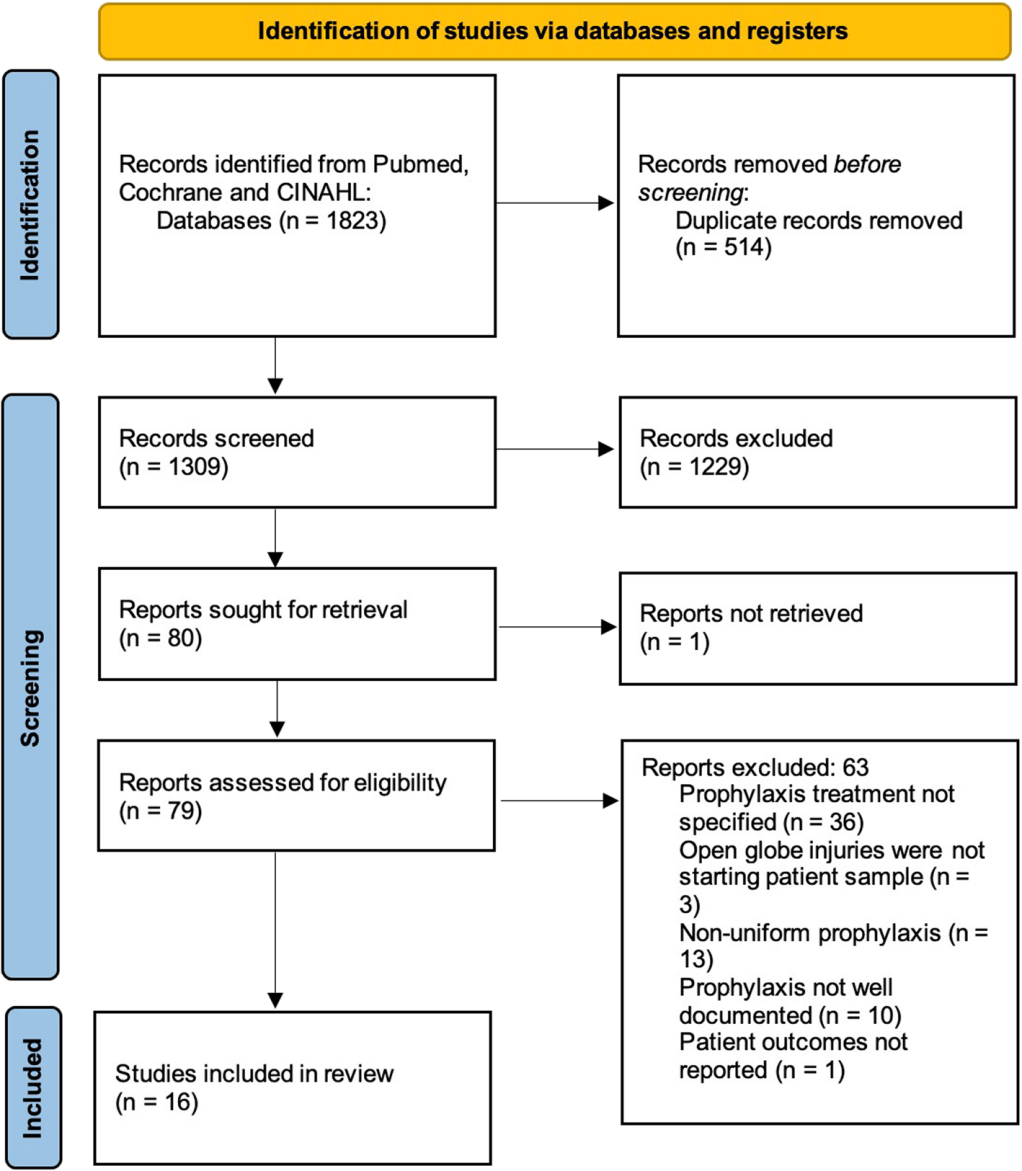
PRISMA Flow Chart of Study Selection

All studies that specified uniform endophthalmitis prophylaxis strategies for patients with OGIs were included. Double- or single-blinded randomized controlled trials and randomized comparison trials, non-randomized controlled trials, and prospective or retrospective observational studies were considered for inclusion. Exclusion criteria included unspecified prophylaxis treatment, starting samples other than OGIs, non-uniform prophylaxis, and prophylaxis not well documented. The remaining exclusion criteria included non-English language, review articles, non-human studies, case reports with less than five patients, duplicates, and inaccessible articles. Two reviewers (J.M.V and M.M.A.) independently screened titles and abstracts to identify all articles that met the inclusion criteria; then full texts of these articles were read to assess which articles would be included in the final analysis. A third reviewer (W.K.M.) resolved any conflicts between reviewers. The Oxford Center for Evidence-Based Medicine criteria was used to critically evaluate the level of evidence of all articles included [6]. The Cochrane Handbook for Systematic Reviews of Interventions version 6.0 was used to assess risk of bias [7]. To evaluate the risk of bias for each study the Risk of Bias in Non-Randomized Studies of Interventions (ROBINS-I) tool was used for each aspect outlined in Fig. 1, bias was graded as low, high, or unclear. J.M.V and M.M.A. independently performed a risk of bias assessment on all studies and compared results for consistency of assessment; disagreements were resolved through discussion with W.K.M.

### Data extraction

Two authors (J.M.V and M.M.A) performed data extraction independently and compared results for accuracy. Extracted data included all information reported in Table 1, the study name, patient demographics, country of origin, total number of patients receiving prophylaxis, prophylaxis management protocol, and the total number of patients in each group who developed endophthalmitis.

**Table 1:** Included Studies Characteristics

Study (ref)	Place	OLE	Study Design	Patients (No.)	Eyes (No.)	Female (%)	Age (Yr.)		
							**Median**	**SD**	**Range**
Abouammoh et al*.*, 2018 [8]	SA	4	R	994	994	12.5	25.7	8.6	0.75–70
Al-Mezaine et al*.*, 2010 [9]	SA	4	R	629	629	12.6	25	14.8	-
Andreoli et al*.*, 2009 [10]	USA	4	R	558	558	19.2	40.1	-	1–95
Dehghani et al*.*, 2018 [11]	Iran	4	R	918	918	13.6	35.3	15.8	-
Duch-Samper et al., 1997 [12]	Spain	4	R	403	403	-	-	-	-
Du Toit et al., 2017 [25]	Russia	2b	P	300	300	23.3	-	-	-
Gupta et al*.*, 2010 [13]	India	2b	P	174	174	48	21.6	-	-
Huang et al*.*, 2016 [14]	USA	2b	R	224	224	18.8	35	22	-
Mansouri et al*.*, 2009 [15]	Iran	4	R	2340	2340	18.6	22.4	16.7	0.33–90
Nakayama et al*.,* 2019 [16]	Brazil	4	R	453	453	42.3	15.6	-	-
Narang et al*.*, 2003 [17]	India	2b	P	70	70	-	-	-	-
Rafati et al*.*, 2013 [18]	Iran	2b	P	111	111	17.1	22.7	12.7	-
Soheilian et al*.*, 2007 [19]	Iran	2b	P	346	346	13.6	-	-	-
Soliman et al*.*, 2008 [20]	Egypt	4	P	147	153	20	22	-	0.17–76
Tabatabaei et al*.*, 2016 [21]	Iran	4	P	1255	1255	26.5	46	19.9	2–98
Verbraeken et al*.*, 1994 [22]	Belgium	4	R	615	615	-	-	-	-

When necessary, data reported in two separate cohorts were combined into a single group for this data table

*Abbreviations: OLE* Oxford Centre for Evidence-Based Medicine Level of Evidence, *No.* Number, *Yr.* Year, *SD* Standard Deviation, *SA* Saudi Arabia, *USA* United States of America, *R* Retrospective, *P* Prospective

### Data analysis

Mixed effects meta-regression models were constructed to investigate the effect of prophylactic regimens, treatment duration, and treatment administration on the rate of endophthalmitis. Summary statistics shown in Supplemental Tables 1, 2 and 3 based on variables shown in Table 2 demonstrated that there were combinations of days, administrations, and regimens that were not observed in the data. The three individual models were constructed in lieu of a model which contained all covariates of interest due to the number of unobserved combinations of the covariates. IOFB will be included in each of these models to control for potential confounding. Analyses were completed using the rma function from the metafor R package and R v.4.0.3 [23, 24].

**Table 2:** Endophthalmitis Rate and Contributing Factors in the Included Studies

Study (ref)	Antibiotic(s)	Route	Treatment Duration (days)	Endoph -thalmitis (%)	IOFB (%)	Presentation < 24 h (%)	Lens Injury (%)
Abouammoh et al*.*, 2018 [8]	V/C V/C	IV IV/IO	5 5	3.74 1.70	13.3 19.3	88.1 89.8	29.6 27.2
Al-Mezaine et al*.*, 2010 [9]	V/C	IV	5	3.50	1.9	88.2	28.9
Andreoli et al*.*, 2009 [10]	V/C	IV	2	0.90	17.0	-	-
Dehghani et al*.*, 2018 [11]	C/A Cip	IV Oral	1 3	2.02 3.66	16.1 20.5	-	-
Duch-Samper et al., 1997 [12]	C/A	IV	3	4.22	-	-	-
Du Toit et al., 2017 [25]	C C	IV Oral	3 3	2.00 2.67	0 0	-	-
Gupta et al*.*, 2010 [13]	Cip	IV	5	11.49	5.7	-	-
Huang et al*.*, 2016 [14]	V/C	IV	2	0.89	20.1	-	25.9
Mansouri et al*.*, 2009 [15]	C/A	IV	3	5.00	24.0	-	-
Nakayama et al*.,* 2019 [16]	C/A	-	-	6.62	-	-	-
Narang et al*.*, 2003 [17]	Cip V/C	IV IV/IO	3 3	18.42 6.25	21.1 25.0	- -	75.0 14.4
Rafati et al*.*, 2013 [18]	C/A	IV	5	4.50	18.9	-	30.5
Soheilian et al*.*, 2007 [19]	C/A C/A	IV IV/IO	5 5	4.79 0.56	15.0 15.1	-	-
Soliman et al*.*, 2008 [20]	V/C	IV/IO	5	6.54	14.4	-	-
Tabatabaei et al*.*, 2016 [21]	V/C V/C	IV Oral	3 3	2.14 2.16	4.30 -	-	-
Verbraeken et al*.*, 1994 [22]	C/A	IV	1	4.07	37.7	-	-

*Abbreviations: IOFB* Intraocular foreign body, *h* hour(s), *V* Vancomycin, *C* Cephalosporin, *A* Aminoglycoside, *Cip* Ciprofloxacin, *IV* Intravenous, *IO* Intraocular

## Results

### Search results and study characteristics

**Fig. 2:**
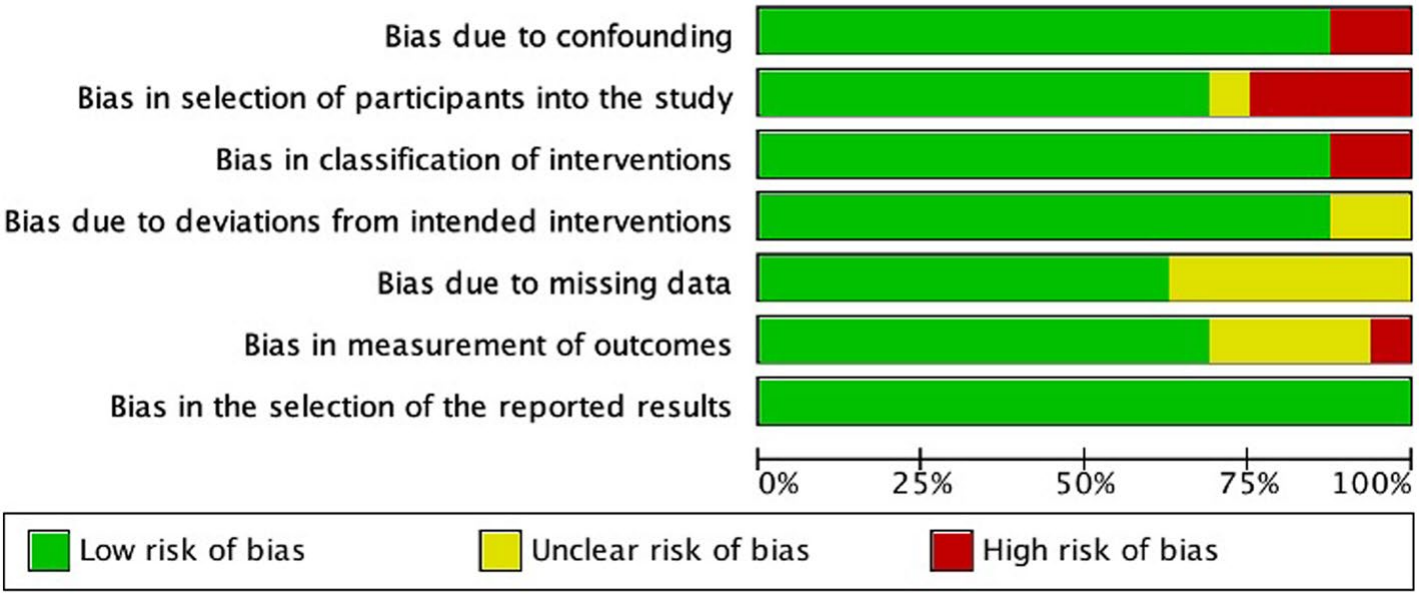
Risk of Bias of Included Studies

The literature search yielded 1823 studies and 16 studies [8–22, 25] were included in the final analysis. A total of 1229 studies were excluded due to meeting exclusion criteria or lacking the necessary factors for inclusion. A diagram outlining the full search process is included in Fig. 1. One included study was a randomized controlled trial [25], 5 were cohort studies [13, 14, 17–19], and 10 were case series [8–12, 15–22], which were level 1b, 2b and 4, respectively according to the Oxford Level of Evidence [26]. Critical appraisal of studies indicated an acceptably low risk of bias for most studies (Fig. 2). Potential sources of bias were most pronounced in bias due to selection of participants into the study. This was most often due to strict exclusion criteria. Our studies were published between 1994 and 2019 and were from 10 different countries. A total of 8,582 patients and 9,543 eyes were included from all the studies. A total of 358 patients from all included studies developed endophthalmitis.

### Effect of prophylactic regimens

Our search strategy yielded the following groups of antibiotic regimens for meta-analysis: ≥ 3^rd^ generation cephalosporin (ceftazidime or cefepime) + vancomycin (regimen 1), 1^st^ generation cephalosporin (cefalotin or cefazolin) + aminoglycoside (gentamicin or netilmicin) (regimen 2), ciprofloxacin monotherapy (regimen 3), and unspecified cephalosporin monotherapy (regimen 4). Table 3 shows the results for the regimen model. The parameter estimates for this model show that, relative to treatment regimen 1, regimens 2 and 4 do not significantly alter the odds of outcome (*p*-values: 0.532 and 0.621, and CI’s (confidence intervals): -0.579–1.121 and -0.698–1.170, respectively). The parameter estimate for regimen 3 indicates that, relative to regimen 1, there is a significant increase in the odds of outcome (*p*-value: 0.000 and CI: 0.597–2.348). Specifically, the parameter estimate for regimen 3 is 1.472, which indicates that the odds of outcome for patients’ treatment under regimen 3 relative to regimen 1 is 4.36 (exp (1.472)), which represents a greater than fourfold increase in the odds of outcome. IOFB was not a significant predictor of outcome.

**Table 3:** Model Results for Treatment Regimen

	**Parameter Estimate**	**CI**	***P*-Value**	**Cases**
**Intercept**	-3.788	-4.303 to -3.273	0.000	88
**Regimen 2**	0.271	-0.579 to 1.121	0.532	191
**Regimen 3**	1.472	0.597 to 2.348	0.000*	37
**Regimen 4**	0.236	-0.698 to 1.170	0.621	32
**IOFB**	0.001	-0.002 to 0.003	0.502	-

*CI* Confidence interval

^*^Statistically significant at 5% level of significance

### Effect of treatment duration

Table 4 shows the results for the days model. The parameter estimates for this model show that, relative to 1 day of treatment, treatment durations of 2, 3 and 5 days do not significantly alter the odds of outcome (*p*-values: 0.139, 0.470, and 0.486, CI’s: -2.724–0.379, -0.708–1.536, and -0.739–1.555, respectively). IOFB was not a significant predictor of outcome.

**Table 4:** Model Results for Treatment Duration

	**Parameter Estimate**	**CI**	***P*-Value**	**Cases**
**Intercept**	-3.555	-4.610 to -2.501	0.000	38
**2 Days**	-1.172	-2.724 to 0.379	0.139	7
**3 Days**	0.414	-0.708 to 1.536	0.470	187
**5 Days**	0.408	-0.739 to 1.555	0.486	96
**IOFB**	0.000	-0.002 to 0.003	0.834	-

*CI* Confidence interval

### Effect of treatment administration

In the included studies, we found oral (type 1), intravenous (IV) (type 2), and IV plus intraocular (type 3) antibiotic administration utilized. Table 5 shows the results for the administration model. The parameter estimates for this model show that, relative to administration type 1, administration types 2 and 3 do not significantly alter the odds of outcome (*p*-values: 0.831 and 0.942, CI’s: -1.194–1.485, -1.626–1.510, respectively). Again, IOFB was not a significant predictor of outcome.

**Table 5:** Model Results for Treatment Administration

	**Parameter Estimate**	**CI**	***P*-Value**	**Cases**
**Intercept**	-3.418	-4.657 to 2.180	0.000	27
**Administration 2**	0.145	-1.194 to 1.485	0.831	282
**Administration 3**	-0.058	-1.626 to 1.510	0.942	19
**IOFB**	0.000	-0.003 to 0.003	0.990	-

*CI* Confidence interval

## Discussion

### Duration of antibiotic administration

Prophylaxis of varying durations is used for prevention of endophthalmitis. The American College of Surgeons and the Surgical infection society found that antimicrobials should be discontinued after skin closure for most procedures; however, this has not been well studied for OGI repair [27]. The risks of prolonged prophylactic regimens are also unclear but prior studies have shown that antibiotics are a leading cause of adverse drug events and emergency department visits [28]. Our analysis was not able to conclude that durations of prophylaxis longer than 1 day achieved statistically better results. Studies with larger samples of patients may be needed to more conclusively elucidate this relationship. However, if longer durations were of little value, then the risks of antibiotic administration > 24 h could outweigh the theoretical benefit.

### Method of antibiotic administration

Although their penetration of the blood-retinal barrier is poor, systemic antibiotics are generally used for endophthalmitis prophylaxis after OGI though there is little agreement on the route of administration [29]. When possible, using oral instead of IV antibiotics can reduce adverse drug-related events and decrease costs [30, 31]. Further, various studies recommend intravitreal antibiotics for high-risk scenarios cases [32–35] though robust clinical evidence for this is lacking. Our results did not find a statistically significant difference in outcomes when IV antibiotics were administrated instead of the oral forms. Similarly, we did not find prophylaxis protocols that included the use of intraocular antimicrobials in severe cases to achieve statistically superior results. Due to the relatively small numbers of total patients in studies evaluating oral antibiotic and intraocular microbial strategies, more studies investigating these methods are needed for meta-analysis to evaluate this aspect of endophthalmitis prophylaxis more definitively. However, if this lack of superiority is found to be true, then oral antibiotics alone could be a reasonable prophylaxis protocol for OGI.

### Prophylactic antibiotic regimen

We compared common antibiotic regimens used to prevent endophthalmitis following OGI. While the success of most regimens did not significantly differ, using ciprofloxacin monotherapy was shown to be significantly less successful than utilizing ≥ 3^rd^ generation cephalosporins with vancomycin. Based on these results, using any of the studied regimens besides ciprofloxacin monotherapy for endophthalmitis prophylaxis is a reasonable approach. However, the choice of an antibiotic regimen for post-traumatic endophthalmitis prophylaxis must consider microbial coverage as a function of spectrum and intraocular bioavailability, along with patient-specific characteristics. As intraocular penetration, antibiotic coverage and patient specific side effects are beyond the scope of this study, future studies can compare the risks and benefits among the three regimens found to have similar efficacy in this study.

While other regimens were used in the literature including levofloxacin and moxifloxacin, unfortunately, there was an insufficient number to allow for inclusion in the meta-analysis. Indeed, previous reviews have recommended levofloxacin and moxifloxacin for prophylaxis of OGIs due to their wide coverage and high intraocular bioavailability in the uninflamed eye [36–38]. Yet, neither of these medications are as active as ciprofloxacin against pseudomonas [29, 39, 40].

### Timing of treatment

Time to treatment has been described as a major factor in endophthalmitis development following ocular trauma and could be more important than prophylactic regimen [9]. Indeed, Dehghani et al. [11] report no significant difference in endophthalmitis risk between antibiotic regimens when presentation following ocular trauma is delayed. Unfortunately, we could not include timing of treatment in our analysis due to the variability in how studies reported this variable; however, data was extracted, reported in Table 6, and reviewed in this section. Thus, please note that Table 6 is merely a review of literature and it not part of the meta-analysis in this study.

**Table 6:** Timing of Treatment in Included Studies

Author(s)	Variable(s)	Endophthalmitis	*P*-value
Al-Mezaine et al*.* 2010 [9]	Interval between trauma and presentation ≤ 24 h (*n* = 555)	7 (1.26%)	0.008*
	Interval between trauma and presentation > 24 h (*n* = 74)	5 (6.76%)	
Andreoli et al*.* 2009 [10]	Time from injury to presentation ≤ 5 h (*n* = 229)	4 (1.75%)	0.19
	Time from injury to presentation > 5 h (*n* = 229)	1 (0.4%)	
	Time from injury to surgical repair ≤ 12 h (*n* = 229)	1 (0.5%)	0.65
	Time from injury to surgical repair > 12 h (*n* = 229)	4 (1.4%)	
Huang et al*.* 2016 [14]	Time from injury to presentation > 48 h (*n* = 15)	4 (26.7%)	0.0002*
	Time from injury to presentation < 48 h (*n* = 207)	1 (0.483%)	
	Time from injury to globe repair > 48 h (*n* = 17)	4 (23.5%)	0.0003*
	Time from injury to globe repair < 48 h (*n* = 205)	1 (0.488%)	

*Abbreviations: h* hour(s), *d* day(s)

^*^Statistically significant at 5% level of significance

Tabatabaei et al. [21] report a trend towards increased time between trauma and surgery for those who developed endophthalmitis (16 ± 3 [Mean ± SD; hours]) with respect to all study participants (13 ± 5). Other authors report timing of treatment categorically for patients who do and do not develop post-traumatic endophthalmitis (Table 6). Huang et al. [14] found a significant increase in the development of endophthalmitis when the interval between injury and presentation was greater than 48 h. Al-Mezaine et al. [9] report a similar finding when presentation is greater than 24 h. However, Andreoli et al. [10] did not find that presenting later than 5 h put patients at a higher risk. When considering the interval between injury and surgical repair, delays greater than 48 h were shown to be significant but waiting 12 h to perform the surgery was not. Collectively, these studies designate delayed treatment as a key element in endophthalmitis development.

### Other influencing factors

In addition to time to presentation and treatment, the success of endophthalmitis prophylaxis is linked to other influencing factors. Variables such as lens disruption, wound cleanliness, rural setting, and intraocular foreign bodies could all contribute to a worse prognosis, even with a standardized prophylactic regimen. Both Sabaci et al. [41] and Thompson et al. [42] found an increased prevalence of endophthalmitis among patients with lens disruption. Additionally, cleanliness of the wound as well as the rural setting are associated with increased risk, primarily due to the presence of more virulent bacteria such as Bacillus [4]. Unfortunately, the frequency of IOFB was the only risk factor reported frequently enough to consider in our analysis and we found that it was not significantly different among any of the comparison groups. Notably, some studies combine other risk factors with delayed treatment to discover particularly high-risk groups. For example, Al-Mezaine et al. [9] report an increased endophthalmitis risk when patients who presented 24 h after injury also had a rural address or intraocular foreign body.

### Limitations

This meta-analysis included 9 retrospective and 7 prospective studies. The majority of these are either case series or cohort studies, with one randomized control trial [25] that studied the effect of just two different antibiotic prophylaxis regimens. As confounders are a major concern with trauma cases in which randomization and controlling are difficult to perform, outcomes may not be representative of the intended investigations. Although these studies came from 9 countries, only articles in the English language were included in the present paper. Additionally, our analysis is limited to the existing literature, which means that our sample may not represent the true population of open globe injuries. Moreover, some methods of endophthalmitis prophylaxis were much more commonly done, which left smaller sample sizes available from which to compare strategies that differed from the conventional approach; this phenomenon yielded less certainty to the negative findings of our study. Finally, the variability in how factors were reported in each article did not allow for analysis of some variables, which could be confounding factors.

## Conclusion

Post-traumatic endophthalmitis is an uncommon complication of open globe injury that often has poor outcomes. Although open globe injuries are a common ocular emergency, prophylactic strategies to prevent endophthalmitis are non-uniform across practices, a reflection of the overall lack of large multicenter randomized control trials. This study aimed to address this gap by utilizing data extracted from systematic review and employing meta-analysis to elucidate significant differences. We addressed the time course, route, and choice of antibiotics while highlighting prognostic factors that should be considered when evaluating patients with ocular trauma. In our meta-analysis, we found ciprofloxacin monotherapy to perform significantly worse than other antibiotics. No specific time course or route of antibiotic administration was significantly associated with better outcomes. However, in our systematic review, we found that in many studies, earlier OGI presentation and treatment was significantly associated with better outcomes. These findings can help guide future studies and treatment protocols utilized for endophthalmitis prophylaxis in open globe injuries.

## Supplementary Information

**Additional file 1: Appendix A.** Search Strategies.

**Additional file 2: Supplemental Table 1.** 2x2 Table of Treatment Durations and Administrations.

**Additional file 3: Supplemental Table 2.** 2x2 Table of Treatment Durations and Regimens.

**Additional file 4: Supplemental Table 3.** 2x2 Table of Treatment Regimens and Administrations.

## Data Availability

The datasets used and/or analysed during the current study are available from the corresponding author on reasonable request.

## Abbreviations

OGI: Open globe injury
IOFB: Intraocular foreign body
IV: Intravenous
