# Supplementary material for: Post-traumatic endophthalmitis prophylaxis: a systematic review and meta-analysis
Source: J Ophthalmic Inflamm Infect. 2022 Nov 18;12:39. doi: 10.1186/s12348-022-00317-y (PMC9672185; doi:10.1186/s12348-022-00317-y)
Supplement: Supplementary file 1 — Additional file 1: Appendix A. Search Strategies. [file 12348_2022_317_MOESM1_ESM.docx]

**Appendix A: Search Strategies**

*PubMed (NLM NIH):*

Search strategy: endophthalmitis AND ("open globe" OR trauma OR traumatic)

Results: 1,579 *(5/18/22)*

*Cochrane Library (Wiley):*

Search strategy:

#1: endophthalmitis (910)

#2: "open globe" (41)

#3: trauma (21304)

#4: traumatic (16070)

#5: #1 AND (#2 OR #3 OR #4) (87)

Results: 87 *(5/18/22)*

*CINAHL (EBSCO):*

Search strategy: endophthalmitis AND ("open globe" OR trauma OR traumatic)

Results: 157 *(5/18/22)*
