# Supplementary material for: Post-traumatic endophthalmitis prophylaxis: a systematic review and meta-analysis
Source: J Ophthalmic Inflamm Infect. 2022 Nov 18;12:39. doi: 10.1186/s12348-022-00317-y (PMC9672185; doi:10.1186/s12348-022-00317-y)
Supplement: Supplementary file 2 — Additional file 2: Supplemental Table 1. 2x2 Table of Treatment Durations and Administrations. [file 12348_2022_317_MOESM2_ESM.docx]

Supplemental Table 1: 2x2 Table of Treatment Durations and Administrations

|  | **Administration 1** | **Administration 2** | **Administration 3** |
| --- | --- | --- | --- |
| **Days 1** | 2 | 0 | 0 |
| **Days 2** | 2 | 0 | 0 |
| **Days 3** | 5 | 1 | 3 |
| **Days 5** | 5 | 3 | 0 |
