# Supplementary material for: Post-traumatic endophthalmitis prophylaxis: a systematic review and meta-analysis
Source: J Ophthalmic Inflamm Infect. 2022 Nov 18;12:39. doi: 10.1186/s12348-022-00317-y (PMC9672185; doi:10.1186/s12348-022-00317-y)
Supplement: Supplementary file 3 — Additional file 3: Supplemental Table 2. 2x2 Table of Treatment Durations and Regimens. [file 12348_2022_317_MOESM3_ESM.docx]

Supplemental Table 2: 2x2 Table of Treatment Durations and Regimens

|  | **Regimen 1** | **Regimen 2** | **Regimen 3** | **Regimen 4** |
| --- | --- | --- | --- | --- |
| **Days 1** | 0 | 1 | 0 | 1 |
| **Days 2** | 2 | 0 | 0 | 0 |
| **Days 3** | 3 | 2 | 2 | 2 |
| **Days 5** | 3 | 3 | 1 | 0 |
