# Supplementary material for: Post-traumatic endophthalmitis prophylaxis: a systematic review and meta-analysis
Source: J Ophthalmic Inflamm Infect. 2022 Nov 18;12:39. doi: 10.1186/s12348-022-00317-y (PMC9672185; doi:10.1186/s12348-022-00317-y)
Supplement: Supplementary file 4 — Additional file 4: Supplemental Table 3. 2x2 Table of Treatment Regimens and Administrations. [file 12348_2022_317_MOESM4_ESM.docx]

Supplemental Table 3: 2x2 Table of Treatment Regimens and Administrations

|  | **Regimen 1** | **Regimen 2** | **Regimen 3** | **Regimen 4** |
| --- | --- | --- | --- | --- |
| **Administration 1** | 5 | 5 | 2 | 2 |
| **Administration 2** | 2 | 1 | 0 | 0 |
| **Administration 3** | 1 | 0 | 1 | 1 |
